# Implementing computerised Aboriginal and Torres Strait Islander health checks in primary care for clinical care and research: a process evaluation

**DOI:** 10.1186/1472-6947-13-108

**Published:** 2013-09-21

**Authors:** Geoffrey KP Spurling, Deborah A Askew, Philip J Schluter, Noel E Hayman

**Affiliations:** 1 Discipline of General Practice, The University of Queensland, Level 8, Health Sciences Building, Building 16/ 910, Royal Brisbane & Women’s Hospital, Herston, Qld 4029 Brisbane, Australia; 2 Inala Indigenous Health Service, Queensland Health, 37 Wirraway Pde, Inala, Qld 4077 Brisbane, Australia; 3 School of Health Sciences, University of Canterbury, Christchurch 8140 New Zealand; 4 School of Nursing and Midwifery, The University of Queensland, Brisbane, Qld 4072, Australia; 5 School of Medicine, The University of Queensland, St Lucia, Qld, 4072 Brisbane, Australia

**Keywords:** Computerised medical record systems, Aboriginal and Torres Strait Islander health, Primary health care

## Abstract

**Background:**

Paper-based Aboriginal and Torres Strait Islander health checks have promoted a preventive approach to primary care and provided data to support research at the Inala Indigenous Health Service, south-west Brisbane, Australia. Concerns about the limitations of paper-based health checks prompted us to change to a computerised system to realise potential benefits for clinical services and research capability. We describe the rationale, implementation and anticipated benefits of computerised Aboriginal and Torres Strait Islander health checks in one primary health care setting.

**Methods:**

In May 2010, the Inala Indigenous Health Service commenced a project to computerise Aboriginal and Torres Strait Islander child, adult, diabetic, and antenatal health checks. The computerised health checks were launched in September 2010 and then evaluated for staff satisfaction, research consent rate and uptake. Ethical approval for health check data to be used for research purposes was granted in December 2010.

**Results:**

Three months after the September 2010 launch date, all but two health checks (378 out of 380, 99.5%) had been completed using the computerised system. Staff gave the system a median mark of 8 out of 10 (range 5-9), where 10 represented the highest level of overall satisfaction. By September 2011, 1099 child and adult health checks, 138 annual diabetic checks and 52 of the newly introduced antenatal checks had been completed. These numbers of computerised health checks are greater than for the previous year (2010) of paper-based health checks with a risk difference of 0.07 (95% confidence interval 0.05, 0.10). Additionally, two research projects based on computerised health check data were underway.

**Conclusions:**

The Inala Indigenous Health Service has demonstrated that moving from paper-based Aboriginal and Torres Strait Islander health checks to a system using computerised health checks is feasible and can facilitate research. We expect computerised health checks will improve clinical care and continue to enable research projects using validated data, reflecting the local Aboriginal and Torres Strait Islander community’s priorities.

## Background

In Australia, improved clinical systems and research opportunities in urban Aboriginal and Torres Strait Islander primary health care are likely to be required to help meet the Australian Government’s ambitious target to close the gap in life expectancy between Indigenous and non-Indigenous Australians within a generation [1]. Aboriginal and Torres Strait Islander peoples living in non-remote locations contribute approximately 60% of the 11 year gap in life expectancy [2], yet a systematic review published in 2010 found only 63 (11%) of 555 articles on Indigenous health published between 2004 and 2009 included information about Aboriginal and Torres Strait Islander peoples from urban areas [3].

In the effort to close the life expectancy gap, an important clinical tool for primary care is the Australian Government funded annual well-person’s health check (HC) for Aboriginal and Torres Strait Islander peoples of all ages. These checks were intended to increase preventive health opportunities, detect chronic disease risk factors and reduce inequities in access to primary care for Aboriginal and Torres Strait Islander peoples [4]. At the Inala Indigenous Health Service (IIHS), we aim to deliver an Aboriginal and Torres Strait Islander HC to all regular patients each year. The evidence for this practice in vulnerable populations is lacking. A systematic review of HCs conducted in primary care settings found improved delivery of recommended clinical preventive services and the authors felt this justified their continued implementation [5]. However, a more recent systematic review published in 2012 by Krogsboll and colleagues reported that general HCs for well adults conducted in primary care or the community were not beneficial and did not reduce morbidity, mortality, nor disease specific mortality. The inclusion criteria for this review excluded older people, children and studies looking specifically at risk factors (such as hypertension) or disease (such as diabetes). Additionally, the majority of trials and patients in this systematic review were conducted in the community where participants were invited to participate which the review authors acknowledged could result in selection bias favouring the well [6]. Clinically significant benefits have been found for other groups receiving HCs such as elderly people who benefited through reduced nursing home admissions, reduced falls and improved physical function [7]. While ethnicity was not an exclusion criteria for the most recent systematic review of HCs, all the included studies were conducted in North America or Europe and no information about the impact of health checks on Indigenous populations was presented [6].

For patients with diabetes we aim to deliver the annual diabetic cycle of care for all patients as per national guidelines produced by Diabetes Australia and the Royal Australian College of General Practitioners [8]. The IIHS facilitates these aims with recall, reminder, and alert systems and we have shown that paper-based HC templates can improve our clinic-based preventive health services [9,10]. However, clinical limitations of the paper-based HC included problems with legibility, non-standardised responses, extra administrative work associated with manually scanning the HCs into the patients’ medical record and paper consumption.

Notwithstanding these limitations, we proceeded with paper-based HC research at the IIHS and obtained ethical approval to evaluate data from a cohort of adult and a cohort of child HCs. The resulting research into childhood obesity [11], stressful events in children [12], self-rated health [13], and middle ear disease [14] relied on a research assistant collating paper HCs, deciphering clinicians’ handwriting and then transferring this information to a data spread sheet line by line. We hypothesised that moving from this time-consuming, error-prone, paper-based process to a computer-based health information technology system would address many of these limitations and enhance patient care through better adherence to preventive care guidelines [15].

Information technology experts built the templates within ERIC, a Queensland state government computerised health record information management system already being used in a few Brisbane hospitals and the local community health sector as an electronic medical record. At the IIHS, we made this web-based, password-protected system available only to the clinical user group at the IIHS to protect patient confidentiality. The ERIC system can generate a Microsoft Excel spreadsheet (Microsoft Corporation, Redmond, WA, USA) collating all patient level data from a given computerised HC template for any given time period. When regular Aboriginal and Torres Strait Islander patients present to the IIHS, nursing staff initiate computerised HCs in ERIC, medical staff complete them and administration staff attach the completed computerised HC to the patient’s medical record in the clinic’s practice software (Practix) as a PDF (portable document format). Computerised HCs were launched at the IIHS in September 2010 and consisted of child and adult Aboriginal and Torres Strait Islander HCs, the annual diabetic HC and a newly introduced antenatal HC.

The objective of this study is to describe the introduction of computerised HCs at the IIHS and evaluate their implementation in terms of user satisfaction, uptake, and utility for research purposes.

## Methods

### Describing the introduction of computerised HCs at the IIHS

#### Setting and participants

The IIHS is situated in south-western Brisbane, Australia. In 2011, 4.8% of people living in the suburb of Inala (postcode area 4077 includes Inala and the three surrounding suburbs) identified themselves as being Aboriginal and/or Torres Strait Islander; one of the highest proportions in Brisbane [16]. Inala is in the lowest 10% of Australian postal areas for scores of socioeconomic indices [17]. In a 2007 audit of adult Aboriginal and Torres Strait Islander paper-based HCs at the IIHS, 42% of participants came from the 4077 postcode area, with remaining participants distributed among 50 other postcodes across Queensland and Northern New South Wales [10]. In 2011, the IIHS saw 1909 Aboriginal and Torres Strait Islander adults and 861 Aboriginal and Torres Strait Islander children as regular patients (defined as those who have consulted with the service at least three times in the preceding two years). Within this population there were approximately 40 pregnant women at any one time and 278 patients with diabetes. Measures taken to improve HC uptake at the IIHS such as recall, reminder and alert systems would ideally lead to a complete, consecutive sample of consenting participants in computerised HC research at the IIHS.

### Computerised HC templates and ongoing template enhancement

Computerised HC content was developed according to Medicare requirements (Australia's publicly funded universal health care system), the Australian National guide to a preventive health assessment for Aboriginal and Torres Strait Islander peoples [18], meetings with relevant clinical IIHS staff and our experience with useful questions from the paper-based HC templates. Aboriginal and Torres Strait Islander HCs use age specific templates, divided into child (0 to 4 years, 5 to 14 years) and adult (15 to 54 years, 55+ years) groupings. The computerised templates used at the IIHS and described in this paper are presented as Additional files 1, 2, 3, 4, 5, and 6. All Aboriginal and Torres Strait Islander HCs include information on demography, resilience factors, health risk factors, socioeconomic factors, examination findings, and health interventions [9,10]. Additional questionnaires embedded within adult computerised HCs include the Fagerstrom test for nicotine dependence [19], the alcohol use disorders identification test (AUDIT) screening tool for hazardous alcohol use [20], and Kessler psychological distress scale (K10) [21].

The antenatal template for pregnant women is commenced at the first antenatal visit and is added to during the pregnancy and only completed at the first postnatal visit. This template includes demographic details, pregnancy details, obstetric history, details of each antenatal visit and a summary of important clinical information at the first postnatal visit including delivery details, the baby’s birth weight, and maternal health parameters.

The computerised monitoring tool for patients with diabetes mellitus is completed annually, attracts government funding (the annual diabetic cycle of care), and is usually conducted in conjunction with the annual Aboriginal and Torres Strait Islander HC and a retinal photo to screen for diabetic retinopathy. This template is designed to cover all the important components relevant to monitoring and managing diabetes mellitus in primary care and includes glycaemic index, blood pressure, body mass index, albumin-creatinine ratio, medications and referrals. A computerised retinal photo reporting form is usually completed at the same time and has become standard practice for patients with diabetes after the IIHS conducted a study demonstrating the value of retinal photography for diabetic retinopathy screening in primary care [22].

All variables in the computerised HCs are constrained by predetermined parameters including radio buttons, tick boxes, free text, integers or numbers with defined decimal places. A small number of fields, such as body mass index and expected date of delivery, are automatically calculated from other fields within the computerised template. Each computerised HC also takes important findings and collates them in a self-populating HC summary. When a computerised HC item response indicates a health issue, explanatory text (e.g. “current smoker”) is programmed to appear in a “Health Check Summary” text box towards the end of the template.

An ongoing process of improving the clinical relevance and usefulness of the HCs occurs at the IIHS. Two years following the introduction of computerised HCs, clinic staff were asked for feedback regarding the content of computerised HCs. Using these responses, a small clinical group made recommendations for improving the templates, including the addition of a self-populating “Actions” text box and functionality to compute cardiovascular risk. These changes are being implemented by the Information Technology (IT) department of Queensland Health.

### Evaluation of computerised HCs at IIHS

#### Staff satisfaction, three months post launch (January 2010)

Nurses, doctors and administrative staff were invited via email to participate anonymously in a brief, post-implementation, on-line questionnaire. This survey was developed and conducted by the IT department at Queensland Health and is an indicative rather than definitive study (Additional file 7). This survey included questions about satisfaction with training, IT support, ease of template use, the value of the HCs and overall satisfaction with the newly introduced computerised HCs. Responses were measured on a scale from one to ten, where ten is most satisfied.

### Computerised HC uptake

The proportion of HCs completed using computerised forms was calculated three months post launch (January 2010). The number of computerised HCs completed after 12 months (20^th^ September 2010 to 19^th^ September 2011) was extracted from ERIC and compared with the number of paper-based HCs from the preceding year as a proportion of the regular IIHS practice patients for each year. Data were imported into specialist statistical software package Stata version 10.0 (StataCorp, College Station, Tex, USA) for analysis. Results of these comparative analyses are reported as point estimate risk difference (RD) with 95% confidence intervals (CIs), using the computerised HC data as the reference.

### Research using data from computerised HCs

#### Validity and missing data

At the IIHS, we have a previously validated criterion standard, the One21seventy audits, against which we will compare the data generated by computerised HCs. The One21seventy project, an annual continuous quality improvement process, commenced at the IIHS in 2010 prior to the introduction of computerised HCs [23]. The One21seventy project uses a range of evidence-based audit tools to monitor clinical practice in the prevention and management of chronic diseases, including four specific audit tools that correspond to the same four broad patient groups included in the computerised HCs at the IIHS; child health, preventive health in adults (aged 15-54 years), patients with diabetes, and pregnant women. For each computerised HC, there are between 14 and 25 variables that correspond with variables from the One21seventy audits and will be used in to validate computerised HC data.

Missing data may occur for computerised HC data collected primarily for clinical purposes and may reduce statistical power and study validity. We will evaluate missing computerised HC data and compare missing data from computerised HCs with missing data from paper-based HCs.

### Ethical computerised HC research of relevance to the community

In their National Statement on Ethical Conduct in Human Research, the National Health and Medical Research Council (NHMRC) advises that informed consent at the commencement of involvement in long-standing studies must explain the unspecified and extended nature of that consent [24]. The statement also counsels that consent may have to be re-negotiated, for example when children transition to adulthood. The consent process described here for the use of these computerised HC data for research purposes was supported by the local Inala Elders and approved with full ethical approval to proceed in December 2010 by the Queensland Health Metro South Human Research Ethics Committee at the Princess Alexandra Hospital (PAH HREC) in Brisbane which covers the IIHS (HREC reference number: HREC/10/QPAH/242).

When patients present to the IIHS and are eligible for a computerised HC, they are invited to consent to the ongoing use of their computerised HC data for research purposes. If they agree to participate, patients (or carers of assenting children aged 15 years and younger) are asked to sign the paper based consent form, the research consent box in the computerised HC is checked “Yes” and this populates the relevant “research consent” field in the data extract. Consenting patients and carers do not have to re-sign the consent form when they return for an HC but must give verbal consent each time they present for an episode of care involving a computerised HC. Children returning for a computerised HC at age 15 years will be eligible for their first adult HC and will need to sign a new consent form. Patients who refuse consent are re-invited to participate at subsequent visits. All information regarding patients who have not given research consent is removed from the data extract prior to any research analysis. Participants who do consent to the use of their computerised HC data for research purposes are not, however, consenting to a particular project and while further written consent is unlikely to be required, additional ethical approval is required for each research project using these data.

Researchers, both internal and external to IIHS, can apply to conduct research using computerised HC data by completing a brief statement of research intent and clearly outlining exactly what data are required. It is expected that the research team will involve an IIHS staff member and ideally an Aboriginal or Torres Strait Islander person. Proposals are assessed by the IIHS research committee based on project feasibility, relevance and cultural safety considerations. If approved by the IIHS research committee, the proposal proceeds to the community jury.

The 14 member Inala Aboriginal and Torres Strait Islander Community Jury for Health Research consists of some self-nominated members, some representatives of local organisations such as the local Elders, and some community members chosen to ensure an appropriate spread of ages, gender and ethnicity. The jury, which meets approximately every three months, was set up in 2011 by the IIHS research committee with terms of reference designed to improve community involvement in decisions surrounding research at the IIHS. Through the jury, the IIHS responds to the principles of community involvement in well communicated ethical research outlined in the Australian NHMRC’s Road Map for improving Aboriginal and Torres Strait Islander health through research [25]. The proposal is discussed with the jury by the lead investigator in plain English. If the proposal obtains jury support, the project must then receive appropriate ethical approval from the PAH HREC. Once approved, the research database containing coded re-identifiable data with identifying information removed is released to the research investigators. Research results are reported back to the jury in an oral presentation and in written report which is made available to the Inala Aboriginal and Torres Strait Islander community.

To improve the relevance of computerised HC research to the local Aboriginal and Torres Strait Islander community we will conduct key informant interviews with jury members and other stakeholders working in the Inala Aboriginal and Torres Strait Islander community regarding issues covered by computerised HCs that are of concern or interest to them. The primary outcome of this research will be the identification of the community’s health research priorities which will inform the research agenda of the IIHS into the future.

## Results

### First 3 months: initial staff evaluation

The staff survey at three months received 14 responses (70% response rate). For most measures including overall satisfaction, staff (administrative, nursing and medical users) gave the recently introduced system of computerised HCs a median mark of 8 out of 10 (Table 1). Administrative staff noted that attaching the computerised PDF to the patient’s practice software was significantly easier than scanning in multiple pages of a paper-based HC.

**Table 1:** Satisfaction with implementation and introduction of computerised health checks at the Inala Indigenous Health Service (IIHS) on a scale of 1 to 10 where 1 represents extremely dissatisfied and 10 represents extremely satisfied

**Survey items**	**Median**	**(Min., Max.)**
**Satisfaction with:**		
Consultation during implementation	8	(5, 10)
Training	8	(1, 9)
IT support	8	(5, 10)
Value of computerised health checks to your work	8	(4, 10)
Process of entering information	7.5	(4, 10)
Overall satisfaction	8	(5, 9)

### First 3 months: computerised HC uptake

Three months following the September 2010 launch of computerised HCs, two HCs had been completed using paper forms and 378 using computerised forms out of 380 HCs (99.5% uptake of computerised forms). The lack of integration between the computerised HC platform and IIHS primary health care practice software led to some computerised HCs not being completed as nursing staff no longer physically handed the paper HC to medical staff for completion. Nursing staff now alert medical staff to the presence of a new computerised HC using bright pink text in the patient’s progress notes and this appears to have resolved the communication breakdown.

### First 12 months: computerised HC research consent rate, uptake and evaluation

Approximately 3% of adult patients and 4% of children’s parents did not provide consent for their data to be used in research projects. There were no significant differences between age categories (p = 0.8), ethnicity (p = 0.7) and gender (p = 0.8) of those who consented and those who did not. The total number of patients receiving a computerised HC completed after 12 months (September 2010 to September 2011) compared with paper-based HCs from the preceding year are presented in Table 2. There was a reduction in the proportion of child computerised HCs compared to paper-based HCs and an increase in the proportion of adult computerised HCs compared to paper-based HCs. The overall number of completed computerised HCs as a proportion of regular patients in their first year at the IIHS increased compared to the overall number of paper-based HCs in the preceding year with a risk difference of 0.07 (95% CI; 0.05, 0.10) (Table 2).

**Table 2:** Uptake of computerised health checks (HC) in September 2010-2011 compared to the preceding year of paper-based HCs

	**September 2009-2010**			**September 2010-2011**			
	**Paper HCs**			**Computerised HCs**			**Risk difference (95% CI)**
	**n**	**(%)**	N*	**n**	**%**	N*	
Child HC 0-14 years	296	*36*	831	215	*25*	861	−0.13 (-0.18, -0.07)
Adult HC 15-54 years	419	*29*	1467	752	*47*	1588	0.20 (0.16, 0.24)
Older persons HC 55+ years	99	*31*	317	132	*41*	321	0.11 (0.03, 0.19)
Annual Diabetic Cycle of Care	129	*51*	255	138	*50*	278	−0.01 (-0.09, 0.08)
Retinal Photo Reporting Form	118	*46*	255	115	*41*	278	−0.05 (-0.14, 0.04)
Total	1061	*34*	3125	1352	*41*	3326	0.07 (0.05, 0.10)
Antenatal HC	N/A			52	62	84	

*Regular patients attending the Inala Indigenous Health Service.

### Research using computerised HCs

By September 2011, two research projects had received approval from the community jury and PAH HREC to commence using computerised HC data. One of these projects explores characteristics of the first antenatal visit and lessons that can be learnt from these presentations at the IIHS [26].

## Discussion

The issues of confidentiality, trust and respect that arise with the creation of computerised forms and databases are particularly important to manage in a culturally appropriate way in Aboriginal and Torres Strait Islander communities. We have demonstrated that the implementation of a health information technology system using practice based computerised HCs is feasible in Aboriginal and Torres Strait Islander primary health care.

The number of HCs conducted at the IIHS as a proportion of regular patients increased following the introduction of computerised HCs indicating at least similar clinician usability and acceptance compared to paper-based HCs. We suppose that the shorter form and other benefits of the computerised form such as constrained answers and a summary self-populating problem list have outweighed the challenge posed by a new information technology system. There are a number of clinical advantages to incorporating computerised HCs in primary care. Using computerised Aboriginal and Torres Strait Islander HCs as data collection tools, as far as we know an Australian first, means they represent a clinical intervention with no increased inconvenience for patients. Additionally, fully computerised medical records are less likely to result in missing clinical data than paper-based systems [27]. Reductions in missing clinical data have been associated with a reduction in adverse events in Australian general practice [28] and are likely to improve the quality of research information derived from these clinic data. Cost-effectiveness analysis following the implementation of other computerised health information systems in clinical settings found benefits were likely to outweigh investment costs, though this could take from between 3 to 13 years [29].

The computerised HCs at IIHS collate important findings in a self-populating problem list which is a useful summary for clinicians. Computerised HCs have the potential to use decision support in domains including cardiovascular risk calculation and referral prompts. These types of minimally intrusive point of care reminders have been shown to improve practice quality in other office-based clinical settings [30]. In the future, wireless functionality could enable computerised HC deployment in the home or other clinic settings using mobile technologies and has demonstrated value in hospital settings [31].

For research, the introduction of this health information technology system has facilitated the generation of a large clinical dataset at minimal extra inconvenience to patients. The primary health care setting has the potential to conduct large numbers of computerised HCs over time which will help us respond to calls for research which informs strategies to address the health needs of Aboriginal and Torres Strait Islander peoples living in urban areas in Australia [3].

Limitations exist for research based on consecutive samples where participation is influenced by both patient and clinic factors. In other clinical settings, such as the emergency department, selection bias associated with consecutive samples has been described as being of low clinical relevance [32]. In our first year of implementing computerised Aboriginal and Torres Strait Islander HCs at the IIHS, it is of concern that relatively fewer child HCs were completed. This may have been a result of high staff turnover among child health nurses at the IIHS, lack of room for carrying out child HCs or perhaps the computerised form for children was too long. Future evaluations of computerised HCs would benefit from asking patients as well as clinical staff about the experiences of having a HC. As the clinical services of the IIHS move into a new, larger building in 2013 we expect the capacity to conduct computerised HCs will be greatly enhanced. We also expect developments in our general practice software will lead to better integration of computerised HCs and the patient’s medical record within the one information technology system. Clinic-based populations are likely to be systematically different to the community which will affect the external validity and generalizability of research results [33].

## Conclusions

We expect improvements in patient care owing to the benefits of legibility, completeness, security and decision support for computerised HCs over paper HCs. Future objectives include the integration of computerised HCs within general practice clinical software for use in multiple sites. The research potential of computerised HCs will be further enhanced by data validation and community consultation processes designed to enable credible research relevant to the community.

## Abbreviations

IIHS: Inala indigenous health service; HC: Health check; PDF: Portable document format; PA HREC: Princess Alexandra human research Ethics Committee; NHMRC: National Health and Medical Research Council; NACCHO: National Aboriginal community controlled health organisation.

## Competing interests

GS, DA and NH are all Queensland Health employees. GS and NH are clinicians and researchers using the new computerised templates in this primary health care setting.

## Authors’ contributions

GS conceived of the project, helped design the templates, obtained ethical approval and wrote the article; DA provided advice at all stages of project implementation and provided advice on template design, ethical matters, research planning and helped draft the final manuscript; PS provided advice on ethical matters, data storage/security, research planning and helped draft the final manuscript; NH helped design the templates, provided advice on ethical approval and future research directions. All authors read and approved the final manuscript.

## Pre-publication history

The pre-publication history for this paper can be accessed here:

http://www.biomedcentral.com/1472-6947/13/108/prepub

## Supplementary Material

Additional file 1 Aboriginal and Torres Strait Islander computerised health check template for children aged 0 to 4 years. Click here for file

Additional file 2 Aboriginal and Torres Strait Islander computerised health check template for children aged 5 to 14 years. Click here for file

Additional file 3 Aboriginal and Torres Strait Islander computerised health check template for adults aged 15 to 54 years. Click here for file

Additional file 4 Aboriginal and Torres Strait Islander computerised antenatal health check template. Click here for file

Additional file 5 Aboriginal and Torres Strait Islander computerised diabetes health check template. Click here for file

Additional file 6 Aboriginal and Torres Strait Islander computerised diabetic retinopathy template. Click here for file

Additional file 7 Computerised health check user satisfaction survey. Click here for file
