# Supplementary material for: Implementing computerised Aboriginal and Torres Strait Islander health checks in primary care for clinical care and research: a process evaluation
Source: BMC Med Inform Decis Mak. 2013 Sep 21;13:108. doi: 10.1186/1472-6947-13-108 (PMC3849740; doi:10.1186/1472-6947-13-108)
Supplement: Additional file 1 — Aboriginal and Torres Strait Islander computerised health check template for children aged 0 to 4 years. [file 1472-6947-13-108-S1.pdf]

	Metro South Health Service District<br>Indigenous Health Check: Age 0-4<br>Community Health	
Queensland Government<br>Queensland Health		UR Number<br>Surname<br>Other Names<br>DOB<br>Sex

An * indicates a mandatory field.

A. PATIENT DETAILS

Age: *	
Is there consent by parent/carer to perform health check? *	◯ Yes ◯ No

Consent for health assessment to be used in research *	◯ Yes ◯ No		
Had a previous 715 (0-4) at this practice?	◯ Yes ◯ No ◉ Missing	Is there any new medical and family history?	◯ Yes ◯ No ◉ Missing
Mother's Name:		Mother's Age:	Mother's URN:*<br>◯ Not in ERIC
Father's Name:		Father's Age:	Father's URN:*<br>◯ Not in ERIC

OR

Carer's Name (and relationship to child):	

Sibling Name		Sibling Age:	

Add Siblings

Single Parent:	◯ Yes ◯ No ◉ Missing
Mother Employed:	◯ Yes ◯ No ◯ Unsure ◉ Missing
Father Employed:	◯ Yes ◯ No ◯ Unsure ◉ Missing
Other people involved in the child's care:	

Indigenous Status

Child: *	◯ Aboriginal ◯ Torres Strait ◯ Both ◉ Missing
Mother: *	◯ Aboriginal ◯ Torres Strait ◯ Both ◯ Non-Indigenous ◉ Missing
Father: *	◯ Aboriginal ◯ Torres Strait ◯ Both ◯ Non-Indigenous ◉ Missing

B. PREVIOUS HEALTH CHECKS

Has the patient had a previous Medicare item 715 (0-4) Health Check?	◯ Yes ◯ No ◯ Unsure ◉ Missing
If < 2 months, has the patient received a newborn check?	◯ Yes ◯ No ◯ Unsure ◉ Missing
Has the patient had a paediatric review in the last 12 months	◯ Yes ◯ No ◯ Unsure ◉ Missing

C. IMMUNISATION STATUS

Is the patient's immunisation status up to date?	◯ Yes ◯ No ◯ Unsure ◉ Missing
Comments:	

D. MEDICAL HISTORY

What was the mode of delivery?	◯ Vaginal ◯ Caesarean ◯ Unsure ◉ Missing
At what gestation (weeks) was the first antenatal visit?	**k**
What was the gestation (weeks) at birth?	**k**
What was the birth weight (grams)?	g
Where there any complications during or shortly after delivery?	◯ Yes ◯ No ◉ Missing
If yes please specify:	
Were there any other antenatal/ postnatal issues of concern?	◯ Yes ◯ No ◉ Missing
If yes please specify:	
Did the child receive neonatal screening for hearing?	◯ Yes ◯ No ◯ Unsure ◉ Missing
What was the outcome?	◯ Pass ◯ Fail ◉ Missing
Regular Snoring	◯ Yes ◯ No ◯ Unsure ◉ Missing
Number of URTIs in the past year?	
Any constipation in the past year?	◯ Yes ◯ No ◉ Missing
**Past Medical History**	
Growth faltering/Failure to Thrive:	◯ Yes ◯ No ◯ Unsure ◉ Missing

Recurrent chest infection:	◯ Yes ◯ No ◯ Unsure ◉ Missing
Pneumonia:	◯ Yes ◯ No ◯ Unsure ◉ Missing
Rheumatic Heart Disease:	◯ Yes ◯ No ◯ Unsure ◉ Missing
Asthma:	◯ Yes ◯ No ◯ Unsure ◉ Missing
Ear infections:	◯ Yes ◯ No ◯ Unsure ◉ Missing
Skin infections:	◯ Yes ◯ No ◯ Unsure ◉ Missing
Other:	◯ Yes ◯ No ◯ Unsure ◉ Missing
If Other, please specify - hospitalisations, injuries, burns:	
**Relevant Family Medical History (Mother or Father):**	
Hypertension:	◯ Yes ◯ No ◯ Unsure ◉ Missing
Gestational Diabetes:	◯ Yes ◯ No ◯ Unsure ◉ Missing
CVD:	◯ Yes ◯ No ◯ Unsure ◉ Missing
Mental illness (e.g. depression, anxiety, schizophrenia):	◯ Yes ◯ No ◯ Unsure ◉ Missing
Asthma/Atopy/Eczema:	◯ Yes ◯ No ◯ Unsure ◉ Missing
Other:	◯ Yes ◯ No ◯ Unsure ◉ Missing
Please specify - if yes to any of the above:	
**CHILD**	
**Current Medications (prescriptions and over the counter):**	
**Allergies/drug intolerances:**	

E. SOCIAL HISTORY/CARER CONCERNS

Who does the child live with? (e.g parent, grandparent, friend?)	Parent	◯ Yes ◯ No ◉ Missing
	Siblings	◯ Yes ◯ No ◉ Missing
	Grandparent	◯ Yes ◯ No ◉ Missing
	Foster Parent	◯ Yes ◯ No ◉ Missing

	Friend	○ Yes ○ No ◉ Missing
	Other	○ Yes ○ No ◉ Missing
If Other, please specify:		
Who is the primary carer of the child?		
How many people usually live at the house?		
Does the carer have any concerns about the child's development?	○ Yes ○ No ◉ Missing	
Any concerns about hearing/listening/talking?	○ Yes ○ No ◉ Missing	
Any concerns about vision?	○ Yes ○ No ◉ Missing	
If Yes to any of the above, please specify:		
**Stressful Life Events**		
Have there been any stressful life events to cause you or your child to be upset?	○ Yes ○ No ◉ Missing	
Conflict at home?	○ Yes ○ No ◉ Missing	
Family Deaths?	○ Yes ○ No ◉ Missing	
Exposure to violence?	○ Yes ○ No ◉ Missing	
Illness to primary caregiver?	○ Yes ○ No ◉ Missing	
Abuse?	○ Yes ○ No ◉ Missing	
Comments:		
Are there any concerns about the mother/carer's current well-being?	○ Yes ○ No ◉ Missing	
If Yes, please specify (e.g. support network, stressors, mood, general health)		
**Domestic Violence/Abuse**		
Are you ever afraid of your partner?	○ Yes ○ No ◉ Missing	

In the last year, has your partner kicked, punched or otherwise hurt you?	◯ Yes ◯ No ◉ Missing
In the last year, has your partner threatened to hurt you?	◯ Yes ◯ No ◉ Missing
Do you feel threatened or afraid of anyone in the community?	◯ Yes ◯ No ◉ Missing
Would you like help with any of this now?	◯ Yes ◯ No ◉ Missing
Has the parent experienced abuse as a child? (emotional, neglect, physical, sexual):	◯ Yes ◯ No ◉ Missing
If yes to any of the above please comment as appropriate:	

F. LEARNING AND BEHAVIOUR

Indicate whether the child attends any of the following:	
Playgroup:	◯ Yes ◯ No ◉ Missing
Childcare centre:	◯ Yes ◯ No ◉ Missing
Pre-Prep or Prep:	◯ Yes ◯ No ◉ Missing
Other (please specify):	
Are any of the above indigenous organisations?	◯ Yes ◯ No ◉ Missing

G. NUTRITION

Was the child ever breastfed?	◯ Yes ◯ No ◉ Missing
Is the child currently being breastfed?	◯ Yes ◯ No ◉ Missing
If no, what age was the breastfeeding stopped?	months
Was the child ever bottle fed?	◯ Yes ◯ No ◉ Missing
Is the child currently bottle fed?	◯ Yes ◯ No ◉ Missing
If No, what age was the bottle feeding stopped?	months
What age did the child start cow's milk or dairy food (e.g. custard)?	months
What age did the child start solids?	months
What was their first solid food?	
What is the child's typical diet?	
Breakfast:	
Lunch:	
Dinner:	

Snacks:	
What is the vegetable and fruit component of this diet?	◯ None<br>◯ Less than 5 serves of vegetables and 2 serves of fruit<br>◯ Equal to or more than 5 serves of vegetables and 2 serves of fruit<br>◉ Missing
Since this time yesterday has the baby/child had any of the following?	
Breast milk:	◯ Yes ◯ No ◉ Missing
Baby formula:	◯ Yes ◯ No ◉ Missing
Milk (tin/powder/fresh):	◯ Yes ◯ No ◉ Missing
Flavoured milk:	◯ Yes ◯ No ◉ Missing
Tea:	◯ Yes ◯ No ◉ Missing
Soft drink/cordial:	◯ Yes ◯ No ◉ Missing
Fruit juice:	◯ Yes ◯ No ◉ Missing
Water:	◯ Yes ◯ No ◉ Missing
Take away food:	◯ Yes ◯ No ◉ Missing
Any concerns about feeding?	◯ Yes ◯ No ◉ Missing
If Yes, please specify:	

H. PARENTAL SUBSTANCE USE

**Smoking**	
Does anyone living in the household currently smoke regularly?	◯ Yes ◯ No ◉ Missing
If Yes, does anyone smoke inside the house?	◯ Yes ◯ No ◉ Missing
If Yes, please state the relationship to the child: Mother, Father, Other (please specify)	
If Yes, do they want assistance to quit smoking?	◯ Yes ◯ No ◯ Unsure ◉ Missing

**Alcohol** *Based on current national alcohol guidelines	
Does the child's mother drink alcohol?	◯ Yes ◯ No ◉ Missing
If Yes, please state if harmful or non-harmful	☐ > 2 std drinks/day avg; drinks 6-7 days/wk; > 14 std drinks/wk<br>☐ <= 2 std drinks/day;1-2 alcohol free days/wk; <14 std drinks/wk

levels:	☐ > 4 std drinks on any one day<br>☑ Missing
Does the child's father drink alcohol?	○ Yes ○ No ◉ Missing
If Yes, please state if harmful or non-harmful levels:	☐ > 4 std drinks/day avg or drinks 6-7 days/wk or > 28 std drinks/wk<br>☐ ⩽ 4 std drinks/day, 1-2 alcohol free days/wk or < 28 std drinks/wk<br>☐ > 6 std drinks on any one day<br>☑ Missing

Other Substances

Does a parent/carer use other substances?	○ Yes ○ No ◉ Missing	
Marijuana	○ Yes ○ No ◉ Missing	○ Daily ○ Weekly ○ Monthly ○ Occasionally ◉ Missing
Amphetamines/Ice	○ Yes ○ No ◉ Missing	○ Daily ○ Weekly ○ Monthly ○ Occasionally ◉ Missing
Heroin	○ Yes ○ No ◉ Missing	○ Daily ○ Weekly ○ Monthly ○ Occasionally ◉ Missing
Cocaine	○ Yes ○ No ◉ Missing	○ Daily ○ Weekly ○ Monthly ○ Occasionally ◉ Missing
Inhaled solvents	○ Yes ○ No ◉ Missing	○ Daily ○ Weekly ○ Monthly ○ Occasionally ◉ Missing
Methadone	○ Yes ○ No ◉ Missing	○ Daily ○ Weekly ○ Monthly ○ Occasionally ◉ Missing

Did the mother use any of the following substances during her pregnancy of this child? ○ Yes ○ No ◉ Missing

- ☐ Smoking
- ☐ Alcohol
- ☐ Marijuana
- ☐ Amphetamines
- ☐ Heroin

PART 2: NURSE'S ASSESSMENT
A. CHILDHOOD DEVELOPMENT

Were any problems with gross motor development identified:	○ Yes ○ No ◉ Missing
If yes, comment:	
Were any problems with fine motor development identified:	○ Yes ○ No ◉ Missing
If yes, comment:	

Were any problems with language development identified:	○ Yes ○ No ◉ Missing
If yes, comment:	
Were any problems with social development identified:	○ Yes ○ No ◉ Missing
If yes, comment:	

Assessment Outcome:

○ Satisfactory
○ Recheck (child not able to be adequately assessed)
○ Known deficit
○ Review (concerns/mild delay)
○ Refer
◉ Missing

B. DYNAMICS

Please comment on the interaction between parent/carer and child (if indicated):

Problem identified:	○ Yes ○ No ◉ Missing
Behavioural intervention could be of use:	○ Yes ○ No ◉ Missing
Comment:	

C. PHYSICAL ASSESSMENT

Weight:	**kg**
Height:	**cm**
Head circumference:	**cm**

Oral Health

Are the child's teeth brushed twice daily?	○ Yes ○ No ○ N/A ◉ Missing
If so, who brushes them?	○ Child ○ Parent/Care giver ○ Unsure ◉ Missing
If child brushes their own teeth,<br>are they supervised?	○ Yes ○ No ◉ Missing
Does the child have gum disease?	○ Yes ○ No ◉ Missing
Does the child have dental caries?	○ Yes ○ No ◉ Missing
If yes please specify which stage:	○ Stage 1<br>○ Stage 2<br>○ Stage 3

	◯ Stage 4<br>◉ Missing
Other dental problems (please specify):	
**Nurse's suggestion for doctor to review:**	

PART 3: MEDICAL ASSESSMENT

A. GROWTH

Is there evidence of growth faltering?	◯ Yes ◯ No ◉ Missing

B. PHYSICAL EXAMINATION

**Newborn Examination (0-2 months)**		
Was a full newborn examination performed today?	◯ Yes ◯ No ◉ Missing	
If Yes:	◯ Normal ◯ Abnormal ◉ Missing	
If Abnormal, please specify:		
**Eyes**		
Red reflex present (< 8 weeks)	◯ Yes ◯ No ◉ Missing	
Squint	◯ Yes ◯ No ◉ Missing	
If abnormal, please specify:		
**Ears (Tympanic membrane; TM)**	**L**	**R**
TM Normal	☐	☐
TM obscured by wax	☐	☐
TM dry perforation	☐	☐
TM wet perforation	☐	☐
Tympanic membrane bulging	☐	☐
Other	☐	☐
Please specify:		
**Skin**		
Skin Problems?	◯ Yes ◯ No ◉ Missing	
Impetigo or boils	◯ Yes ◯ No ◉ Missing	

Sores (more than three)	◯ Yes ◯ No ◉ Missing
Scabies	◯ Yes ◯ No ◉ Missing
Ringworm	◯ Yes ◯ No ◉ Missing
Other	◯ Yes ◯ No ◉ Missing
Please specify:	

Heart

Cardiac Auscultation	◯ Normal ◯ Abnormal ◉ Missing
If abnormal, please specify:	

Respiratory

Throat examination	◯ Normal ◯ Abnormal ◉ Missing
If abnormality detected, please specify:	
Chest examination	◯ Normal ◯ Abnormal ◉ Missing
Respiratory illness detected	◯ Yes ◯ No ◉ Missing
If abnormality detected, please specify:	

Abdomen (if indicated)

Abdominal examination	◯ Normal ◯ Abnormal ◉ Missing
If abnormality detected, please specify:	

Other examinations conducted:

How has the child's mood been in the past month?	Happy:	◯ No ◯ A little ◯ A lot ◯ Unsure ◯ N/A ◉ Missing
	So sad nothing could make you happy:	◯ No ◯ A little ◯ A lot ◯ Unsure ◯ N/A ◉ Missing
	So worried or scared you felt sick in the belly:	◯ No ◯ A little ◯ A lot ◯ Unsure ◯ N/A ◉ Missing
	So angry or wild you couldn't walk away and cool down:	◯ No ◯ A little ◯ A lot ◯ Unsure ◯ N/A ◉ Missing
Known Health Problems:	☐ Asthma<br>☐ Rheumatic Heart Disease	

PROBLEM LIST

C. DIAGNOSIS

Was a new diagnosis made?	○ Yes ○ No ◉ Missing
If Yes, specify details:	
**Significant health problems and issues:**	

PART 4: ACTIONS

Was a referral made?	○ Yes ○ No ◉ Missing
If Yes, for which services?	
Was advice given?	○ Yes ○ No ◉ Missing
If Yes, outline?	
Was medication recommended?	○ Yes ○ No ◉ Missing
If Yes, which one(s)?	
Were vaccinations provided during this health check?	○ Yes ○ No ◉ Missing
If Yes, specify details:	
Other intervention?	○ Yes ○ No ◉ Missing
If Yes:	
Date of health check:	20/09/2010

Sign Off

☐ **Completed?** (Select this only when Health Check is complete)

User Name _____ **Password** _____ Save

Health Check Completed by:

Signed:

Date:
