# Supplementary material for: Implementing computerised Aboriginal and Torres Strait Islander health checks in primary care for clinical care and research: a process evaluation
Source: BMC Med Inform Decis Mak. 2013 Sep 21;13:108. doi: 10.1186/1472-6947-13-108 (PMC3849740; doi:10.1186/1472-6947-13-108)
Supplement: Additional file 2 — Aboriginal and Torres Strait Islander computerised health check template for children aged 5 to 14 years. [file 1472-6947-13-108-S2.pdf]

Queensland Government Queensland Health	Metro South Health Service District<br>**Indigenous Health Check: Age 5-14**<br>**Community Health**	UR Number<br>Surname<br>Other Names<br>DOB<br>Sex

An * indicates a mandatory field.

A. PATIENT DETAILS

Age: *	
Is there consent by parent/carer to perform health check? *	◯ Yes ◯ No

Consent for health assessment to be used in research *	◯ Yes ◯ No		
Had a previous 715 (5-14) at this practice?	◯ Yes ◯ No ◉ Missing	Is there any new medical and family history?	◯ Yes ◯ No ◉ Missing
Mother's Name:		Mother's Age:	Mother's URN:<br>◯ Not in ERIC
Father's Name:		Father's Age:	Father's URN:<br>◯ Not in ERIC
**OR**			
Carer's Name (and relationship to child):			
Sibling Name		Sibling Age:	
	Add Siblings		
Single Parent:	◯ Yes ◯ No ◉ Missing		
Mother Employed:	◯ Yes ◯ No ◯ Unsure ◉ Missing		
Father Employed:	◯ Yes ◯ No ◯ Unsure ◉ Missing		
Other people involved in the child's care:			
**Indigenous Status**			
Child: *	◯ Aboriginal ◯ Torres Strait ◯ Both ◉ Missing		
Mother: *	◯ Aboriginal ◯ Torres Strait ◯ Both ◯ Non-Indigenous ◉ Missing		

Father: *	◯ Aboriginal ◯ Torres Strait ◯ Both ◯ Non-Indigenous ◉ Missing

B. PREVIOUS HEALTH CHECKS

Has the patient had a previous Medicare item 715 (5-14) Health Check?	◯ Yes ◯ No ◯ Unsure ◉ Missing
Has the patient had a paediatric review in the last 12 months	◯ Yes ◯ No ◯ Unsure ◉ Missing

C. IMMUNISATION STATUS

Is the patient's immunisation status up to date?	◯ Yes ◯ No ◯ Unsure ◉ Missing
Comments:	

D. MEDICAL HISTORY

**Birth**	
What was the mode of delivery?	◯ Vaginal ◯ Caesarean ◯ Unsure ◉ Missing
What was the gestation (weeks) at birth? *	k
What was the birth weight (grams)? *	**g**
Were there any complications during or shortly after delivery?	◯ Yes ◯ No ◉ Missing
If yes please specify:	
Were there any other antenatal/ postnatal issues of concern?	◯ Yes ◯ No ◉ Missing
If yes please specify:	
Did the child receive neonatal screening for hearing?	◯ Yes ◯ No ◯ Unsure ◉ Missing
What was the outcome?	◯ Pass ◯ Fail ◉ Missing
Regular Snoring	◯ Yes ◯ No ◯ Unsure ◉ Missing
Number of URTIs in the past year?	
Any constipation in the past year?	◯ Yes ◯ No ◉ Missing
**Past Medical History**	
Growth faltering/Failure to Thrive:	◯ Yes ◯ No ◯ Unsure ◉ Missing

Recurrent chest infection:	Yes No Unsure Missing
Pneumonia:	Yes No Unsure Missing
Rheumatic Heart Disease:	Yes No Unsure Missing
Asthma:	Yes No Unsure Missing
Ear infections:	Yes No Unsure Missing
Skin infections:	Yes No Unsure Missing
Other:	Yes No Unsure Missing
If Other, please specify - hospitalisations, injuries, burns:	
**Relevant Family Medical History (Mother or Father):**	
Hypertension:	Yes No Unsure Missing
Gestational Diabetes:	Yes No Unsure Missing
CVD:	Yes No Unsure Missing
Mental illness (e.g. depression, anxiety, schizophrenia):	Yes No Unsure Missing
Asthma/Atopy/Eczema:	Yes No Unsure Missing
Other:	Yes No Unsure Missing
Please specify - if yes to any of the above:	
**CHILD**	
**Current Medications (prescriptions and over the counter):**	
**Allergies/drug intolerances:**	

E. SOCIAL HISTORY/CARER CONCERNS

Who does the child live with? (e.g parent, grandparent, friend?)	Parent	Yes No Missing
	Siblings	Yes No Missing
	Grandparent	Yes No Missing
	Foster Parent	Yes No Missing

	Friend	○ Yes ○ No ◉ Missing
	Other	○ Yes ○ No ◉ Missing
If Other, please specify:		
Who is the primary carer of the child?		
How many people usually live at the house?		
Does the carer have any concerns about the child's development?	○ Yes ○ No ◉ Missing	
Any concerns about hearing?	○ Yes ○ No ◉ Missing	
Any concerns about talking?	○ Yes ○ No ◉ Missing	
Any concerns about vision?	○ Yes ○ No ◉ Missing	
If Yes to any of the above, please specify:		
**Stressful Life Events**		
Have there been any stressful life events to cause you or your child to be upset?	○ Yes ○ No ◉ Missing	
Conflict at home?	○ Yes ○ No ◉ Missing	
Family Deaths?	○ Yes ○ No ◉ Missing	
Exposure to violence?	○ Yes ○ No ◉ Missing	
Illness to primary caregiver?	○ Yes ○ No ◉ Missing	
Abuse?	○ Yes ○ No ◉ Missing	
Comments:		
Are there any concerns about the mother/carer's current well-being?	○ Yes ○ No ◉ Missing	
If Yes, please specify (e.g. support network, stressors, mood, general health)		
**Domestic Violence/Abuse**		
Are you ever afraid of your partner?	○ Yes ○ No ◉ Missing	
In the last year, has your partner kicked, punched or otherwise hurt you?	○ Yes ○ No ◉ Missing	
In the last year, has you partner threatened to hurt you?	○ Yes ○ No ◉ Missing	
Do you feel threatened or afraid of anyone in the	○ Yes ○ No ◉ Missing	

community?	
Would you like help with any of this now?	◯ Yes ◯ No ◉ Missing
Has the parent experienced abuse as a child? (emotional, neglect, physical, sexual):	◯ Yes ◯ No ◉ Missing
If yes, please comment as appropriate:	

F. LEARNING AND BEHAVIOUR

Does the child attend school?	◯ Yes ◯ No ◯ Unsure ◉ Missing
If Yes, what year?	
Does the parent/carer have any concerns about learning?	◯ Yes ◯ No ◉ Missing
If Yes, please specify:	
Does the parent/carer have any concerns about behaviour?	◯ Yes ◯ No ◉ Missing
If Yes, please specify:	
Does the teacher have any concerns about learning or behaviour?	◯ Yes ◯ No ◉ Missing
If Yes, please specify:	
What marks does your child receive on their report card?	◯ Above average<br>◯ Average<br>◯ Below average<br>◯ Unsure<br>◉ Missing
Has the child been suspended / expelled from school	◯ Yes ◯ No ◉ Missing
Comments	
Has the child moved schools in the past 12 months	◯ Yes ◯ No ◉ Missing
If yes, how many times?	

G. NUTRITION

Was the child ever breastfed?	◯ Yes ◯ No ◉ Missing
If Yes, what age was the breastfeeding stopped?	months
Was the child ever bottle fed?	◯ Yes ◯ No ◉ Missing

Is the child currently bottle fed?	◯ Yes ◯ No ◉ Missing
If No, what age was the bottle feeding stopped?	months
What age did the child start solids?	months
What was their first solid food?	
**What is the child's typical diet?**	
Breakfast:	
Lunch:	
Dinner:	
Snacks:	
What is the vegetable and fruit component of this diet?	◯ None<br>◯ Less than 5 serves of vegetables and 2 serves of fruit<br>◯ Equal to or more than 5 serves of vegetables and 2 serves of fruit<br>◉ Missing
How many times in the past week has the child had take away food?	
Since this time yesterday has the baby/child had any of the following?	
Flavoured milk:	◯ Yes ◯ No ◉ Missing
Tea:	◯ Yes ◯ No ◉ Missing
Soft drink/cordial:	◯ Yes ◯ No ◉ Missing
Fruit juice:	◯ Yes ◯ No ◉ Missing
Water:	◯ Yes ◯ No ◉ Missing
Any concerns about diet?	◯ Yes ◯ No ◉ Missing
If Yes, please specify:	
**Activity**	
Did the child exercise / play sport?	◯ Yes ◯ No ◉ Missing
How often in a week is the child physically active?	
A session is > 30 mins exercise that raised their heart rate or caused them to huff and puff.	
How much time (minutes) did the child spend on screen-based media for fun in the previous day (or most recent school day)?	
Screen based media includes watching DVDs, playing video or computer games, internet chatrooms.	
Any other	

comments/observations related to nutrition/physical activity?:	

G. PARENTAL SUBSTANCE USE

**Smoking**	
Does anyone living in the household currently smoke regularly?	◯ Yes ◯ No ◉ Missing
If Yes, does anyone smoke inside the house?	◯ Yes ◯ No ◉ Missing
If Yes, please state the relationship to the child: Mother, Father, Other (please specify)	
If Yes, do they want assistance to quit smoking?	◯ Yes ◯ No ◯ Unsure ◉ Missing
**Alcohol** *Based on current national alcohol guidelines	
Does the child's mother drink alcohol?	◯ Yes ◯ No ◉ Missing
If Yes, please state if harmful or non-harmful levels:	☐ > 2 std drinks/day avg; drinks 6-7 days/wk; > 14 std drinks/wk<br>☐ ⩽ 2 std drinks/day, 1-2 alcohol free days/wk; <14 std drinks/wk<br>☐ > 4 std drinks on any one day<br>☑ Missing
Does the child's father drink alcohol?	◯ Yes ◯ No ◉ Missing
If Yes, please state if harmful or non-harmful levels:	☐ > 4 std drinks/day avg or drinks 6-7 days/wk or > 28 std drinks/wk<br>☐ ⩽ 4 std drinks/day, 1-2 alcohol free days/wk or < 28 std drinks/wk<br>☐ > 6 std drinks on any one day<br>☑ Missing
**Other Substances**	
Does a parent/carer use other substances?	◯ Yes ◯ No ◉ Missing

Marijuana	◯ Yes ◯ No ◉ Missing	◯ Daily ◯ Weekly ◯ Monthly ◯ Occasionally ◉ Missing
Amphetamines/Ice	◯ Yes ◯ No ◉ Missing	◯ Daily ◯ Weekly ◯ Monthly ◯ Occasionally ◉ Missing
Heroin	◯ Yes ◯ No ◉ Missing	◯ Daily ◯ Weekly ◯ Monthly ◯ Occasionally ◉ Missing
Cocaine	◯ Yes ◯ No ◉ Missing	◯ Daily ◯ Weekly ◯ Monthly ◯ Occasionally ◉ Missing
Inhaled solvents	◯ Yes ◯ No ◉ Missing	◯ Daily ◯ Weekly ◯ Monthly ◯ Occasionally ◉ Missing
Methadone	◯ Yes ◯ No ◉ Missing	◯ Daily ◯ Weekly ◯ Monthly ◯ Occasionally ◉ Missing

PART 2: NURSE'S ASSESSMENT
A. CHILDHOOD DEVELOPMENT

Were any problems with gross motor development identified:	◯ Yes ◯ No ◉ Missing
If yes, comment:	
Were any problems with fine motor development identified:	◯ Yes ◯ No ◉ Missing
If yes, comment:	
Were any problems with language development identified:	◯ Yes ◯ No ◉ Missing
If yes, comment:	
Were any problems with social development identified:	◯ Yes ◯ No ◉ Missing
If yes, comment:	

Assessment Outcome:

- ◯ Satisfactory
- ◯ Recheck (child not able to be adequately assessed)
- ◯ Known deficit
- ◯ Review (concerns/mild delay)
- ◯ Refer
- ◉ Missing

B. DYNAMICS

Please comment on the interaction between parent/carer and child (if indicated):

Problem identified:	◯ Yes ◯ No ◉ Missing
Behavioural intervention could be of use:	◯ Yes ◯ No ◉ Missing
Comment:	

C. PHYSICAL ASSESSMENT

Weight:	**kg**
Patient's height:	**cm**
Waist circumference:	**cm** (at umbilicus)
Blood pressure (SDB/DBP):	/ (Systollic/Diastolic)
Pulse rate:	
**Nurse's suggestion for doctor to review:**	

C. VISION

Visual Acuity	◯ With glasses ◯ Without glasses ◉ Missing	R6/ L6/	(Need referral if unable to read 3 symbols on 6/12 line or 2 or more line differences between eyes)

D. ORAL HEALTH

**Oral Health**	
Are the child's teeth brushed twice daily?	◯ Yes ◯ No ◯ N/A ◉ Missing
If so, who brushes them?	◯ Child ◯ Parent/Care giver ◯ Unsure ◉ Missing
If child brushes their own teeth, are they supervised?	◯ Yes ◯ No ◉ Missing
Does the child have gum disease?	◯ Yes ◯ No ◉ Missing
Does the child have dental caries?	◯ Yes ◯ No ◉ Missing
If yes please specify which stage:	◯ Stage 1<br>◯ Stage 2<br>◯ Stage 3<br>◯ Stage 4<br>◉ Missing
Other dental problems (please specify):	

PART 3. MEDICAL ASSESSMENT

A. ADOLESCENT HEALTH (Questions directed to the child if over 10 or less than 10 if appropriate

**Smoking**	
Do you smoke regularly?	◯ Yes ◯ No ◉ Missing
If Yes, how many per day?	
**Alcohol**	
Do you drink alcohol?	◯ Yes ◯ No ◉ Missing
If Yes, how many standard drinks per week?	
Are you concerned that your alcohol intake might be harmful?	◯ Yes ◯ No ◉ Missing
Caffeine (Coffee, tea, cola, iced coffee, V, Red Bull, other):	◯ Yes ◯ No ◉ Missing
Drinks per day:	
**Other Substances**	◯ Yes ◯ No ◉ Missing
Opiates (heroin, methadone, codeine, endone, MS contin)	◯ Yes ◯ No ◉ Missing
Cannabis/Yarndi	◯ Yes ◯ No ◉ Missing

Amphetamines (speed, base, crystal meth, ice, ecstasy, MDMA)	Yes No Missing
Cocaine	Yes No Missing
Hallucinogen (LSD, magic mushrooms)	Yes No Missing
Volatile Substances (paints, glues)	Yes No Missing
Prescription medicine (valium, temazepam)	Yes No Missing
Over the counter medicine (pseudoephedrine)	Yes No Missing
Comments:	

**Sexual and Reproductive Health**	
Are you sexually active?	Yes No Missing
If Yes, do you use protection?	Yes No Missing
If Yes, specify details:	

B. PHYSICAL EXAMINATION

**Eyes**		
Squint	Yes No Missing	
**Ears (Tympanic membrane; TM)**	**L**	**R**
TM Normal	☐	☐
TM obscured by wax	☐	☐
TM dry perforation	☐	☐
TM wet perforation	☐	☐
Tympanic membrane bulging	☐	☐
Other	☐	☐
Please specify:		

Skin Problems?	◯ Yes ◯ No ◉ Missing
Impetigo or boils	◯ Yes ◯ No ◉ Missing
Sores (more than three)	◯ Yes ◯ No ◉ Missing
Scabies	◯ Yes ◯ No ◉ Missing
Ringworm	◯ Yes ◯ No ◉ Missing
Other	◯ Yes ◯ No ◉ Missing
Please specify:	
**Heart**	
Cardiac Auscultation	◯ Normal ◯ Abnormal ◉ Missing
If abnormal, please specify:	
**Respiratory**	
Throat examination	◯ Normal ◯ Abnormal ◉ Missing
If abnormality detected, please specify:	
Chest examination	◯ Normal ◯ Abnormal ◉ Missing
Respiratory illness detected	◯ Yes ◯ No ◉ Missing
If abnormality detected, please specify:	
**Abdomen** (if indicated)	
Abdominal examination	◯ Normal ◯ Abnormal ◉ Missing
If abnormality detected, please specify:	
**Other examinations conducted:**	

How has your mood been over the last month?	Happy?	◯ No ◯ A little ◯ A lot ◯ Unsure ◉ Missing
	So sad nothing could make you happy?	◯ No ◯ A little ◯ A lot ◯ Unsure ◉ Missing
	So worried or scared you felt sick in the belly?	◯ No ◯ A little ◯ A lot ◯ Unsure ◉ Missing
	So angry or wild you could't walk away and cool down?	◯ No ◯ A little ◯ A lot ◯ Unsure ◉ Missing

	Like you wanted to harm yourself?	◯ No ◯ A little ◯ A lot ◯ Unsure ◉ Missing
	Peer relations problems identified	◯ Yes ◯ No ◯ Missing
	Comments:	
	If mental health concerns, please specify:	
Known Health Conditions:	☐ Asthma<br>☐ Rheumatic Heart Disease	

PROBLEM LIST

C. DIAGNOSIS

Was a new diagnosis made?	◯ Yes ◯ No ◉ Missing
If Yes, specify details:	
**Significant health problems and issues:**	

PART 4: ACTIONS

Was a referral made?	◯ Yes ◯ No ◉ Missing
If Yes, outline	
Was advice given?	◯ Yes ◯ No ◉ Missing
If Yes, outline	
Was medication	

recommended?	◯ Yes ◯ No ◉ Missing
If Yes, outline	
Were vaccinations provided during this health check?	◯ Yes ◯ No ◉ Missing
If Yes, outline	
Other intervention?	◯ Yes ◯ No ◉ Missing
If Yes, outline	
Date of health check:	

Health Check Completed by:

Sign Off

☐ **Completed?** (Select this only when Health Check is complete)

User Name ______ **Password** ______ Save

Health Check Completed by:

Signed:

Date:
