# Supplementary material for: Implementing computerised Aboriginal and Torres Strait Islander health checks in primary care for clinical care and research: a process evaluation
Source: BMC Med Inform Decis Mak. 2013 Sep 21;13:108. doi: 10.1186/1472-6947-13-108 (PMC3849740; doi:10.1186/1472-6947-13-108)
Supplement: Additional file 3 — Aboriginal and Torres Strait Islander computerised health check template for adults aged 15 to 54 years. [file 1472-6947-13-108-S3.pdf]

Metro South Health Service District	UR Number	
Indigenous Health Check: Age 15-54	Surname	
Community Health	Other Names	
	DOB	
	Sex	Female

Queensland Government
Queensland Health

An * indicates a mandatory field.

A. PATIENT DETAILS

Date:		
Age:		
Consent for health assessment? *	◯ Yes ◯ No	Date:
Consent for health assessment to be used in research?	◯ Yes ◯ No ◉ Missing	
Previous Health Assessment?	◯ Yes ◯ No ◉ Missing	Date:
Ethnicity: *	◯ Aboriginal ◯ Aboriginal & Torres Strait Islander ◯ Torres Strait Islander ◯ Other ◉ Missing	
Mother's URN:* ◯ Not in ERIC	Father's URN:* ◯ Not in ERIC	

CLINICAL FINDINGS

Allergy	◯ Yes ◯ No ◉ Missing
Which drug/general?	
**Metabolic and Cardiovascular Measures**	
Blood Pressure:	/ mmHg (Systolic/Diastolic)
Pulse:	/min
Weight:	kg
Height:	cm
BMI:	
Waist Measurement:	cm
Blood Glucose Level:	mmol/L
HbA1c	%
**Urinanalysis**	
Glucose:	◯ Negative ◯ Trace ◯ + ◯ ++ ◯ +++ ◉ Missing
Bilirubin:	

	◯ Negative ◯ Trace ◯ + ◯ ++ ◯ +++ ◉ Missing
Ketones:	◯ Negative ◯ Trace ◯ + ◯ ++ ◯ +++ ◉ Missing
Blood:	◯ Negative ◯ Trace ◯ + ◯ ++ ◯ +++ ◉ Missing
Protein:	◯ Negative ◯ Trace ◯ + ◯ ++ ◯ +++ ◉ Missing
Nitrites:	◯ Negative ◯ Positive ◉ Missing
Leucocytes:	◯ Negative ◯ Trace ◯ + ◯ ++ ◯ +++ ◉ Missing
If abnormal proceed to ACR - result:	Albuminuria: ____ mg/L<br>Creatinine: ____<br>AC Ratio: ____

Immunisation History

Immunisation up to date?	◯ Yes ◯ No ◉ Missing	
Gardasil course completed?	◯ Yes ◯ No ◉ Missing	◯ Given ◯ Declined
Fluvax due?	◯ Yes ◯ No ◉ Missing	◯ Given ◯ Declined
Pneumovax due?	◯ Yes ◯ No ◉ Missing	◯ Given ◯ Declined
ADT due?	◯ Yes ◯ No ◉ Missing	◯ Given ◯ Declined
Other due?	◯ Yes ◯ No ◉ Missing	
Please specify:		

Visual Acuity

Best Vision (Use Glasses if available or pinhole):	◯ With glasses	◯ Without glasses	◯ Glasses normally worn but not today ◉ Missing
	**Left Eye**	**Right Eye**	**Both Eyes**
	6/	6/	6/
Identified Problem:			

HEALTH AND LIFESTYLE

Smoking

Never Smoked:	◯ Yes ◯ No ◉ Missing
Ex-smoker:	◯ Yes ◯ No ◉ Missing Year that you quit:
Smoker:	◯ Yes ◯ No ◉ Missing
Wishes to quit:	◯ Yes ◯ No ◉ Missing
Age started:	
Tobacco:	◯ Yes ◯ No ◉ Missing
Number of cigarettes / day:	

Pack Years:	
To complete a Fagerstrom Test, click the following	Fagerstrom Test
Fagerstrom Score:	
Comment:	

Alcohol

Alcohol:	◯ Yes ◯ No ◉ Missing
If yes, please state if harmful or non-harmful levels	☐ > 2 std drinks/day avg; drinks 6-7 days/wk; > 14 std drinks/wk<br>☐ ≤ 2 std drinks/day, 1-2 alcohol free days/wk; <14 std drinks/wk<br>☐ > 4 std drinks on any one day<br>☑ Missing
To complete an Alcohol Screen (Audit), click the following	Alcohol Screen (Audit)
Total audit score:	
Caffeine (coffee, tea, green tea, Red Bull, V drinks, Coke, Pepsi, iced coffee)	◯ Yes ◯ No ◉ Missing
Drinks per day:	

Other Substances

Other Substances	◯ Yes ◯ No ◉ Missing
Opiates (heroine, methadone, codeine, endone, MS contin)	◯ Yes ◯ No ◉ Missing
Cannabis/Yarndi	◯ Yes ◯ No ◉ Missing
Amphetamines (speed, base, crystal meth, ice, ecstacy, MDMA)	◯ Yes ◯ No ◉ Missing
Cocaine	◯ Yes ◯ No ◉ Missing
Hallucinogen (LSD, magic mushrooms)	◯ Yes ◯ No ◉ Missing
Volatile Substances (paints, glues)	◯ Yes ◯ No ◉ Missing
Prescription medicine (valium, temazepam)	◯ Yes ◯ No ◉ Missing
Over the counter medicine	

(pseudoephedrine)	◯ Yes ◯ No ◉ Missing
Comments:	
**Nutrition**	
Are you concerned about your weight?	◯ Yes ◯ No ◉ Missing
Has your weight changed in the past 12 months (are your clothes tighter or looser):	◯ Yes ◯ No ◉ Missing ◯ Tighter ◯ Looser ◉ Missing
Has there been any change in your appetite lately?	◯ I am not hungry/do not feel like eating<br>◯ No change<br>◯ I am always hungry/eating more often<br>◉ Missing
Bowel habits/changes including constipation:	◯ Yes ◯ No ◉ Missing
If Yes, specify:	
Fruit/Vegetable intake in the last 24 hours:	◯ Adequate (2 serves of fruit and 5 vegetables)<br>◯ Sub-optimal<br>◯ None<br>◉ Missing
Take-away (meals per week)	
Soft drink/cordial (glasses per day)	
Identified Nutrition problems:	
**Physical Activity**	
How often in a week are you physically active?	
A session is > 30 mins exercise that raised their heart rate or caused them to huff and puff	
Do you play any regular sport?	◯ Yes ◯ No ◉ Missing
If Yes, please specify:	
Identified problems:	
**Hearing**	
Hearing loss:	◯ Yes ◯ No ◉ Missing
Whisper test done:	◯ Yes ◯ No ◉ Missing
Result:	◯ Heard ◯ Not heard ◉ Missing
Identified problems:	

Actions:	
**Dental**	
Dental Problems:	◯Yes ◯No ◉Missing
Dental Caries:	◯Yes ◯No ◉Missing
Gum Disease:	◯Yes ◯No ◉Missing
Identified Problems:	
**Mental Health**	
Mental Health Issue:	◯Yes ◯No ◉Missing
Select applicable:	
Depression:	◯Yes ◯No ◉Missing *If yes, please do K10*
Suicidal ideas:	◯Yes ◯No ◉Missing *If yes, please do K10*
Anxiety:	◯Yes ◯No ◉Missing *If yes, please do K10*
Psychosis:	◯Yes ◯No ◉Missing
Bipolar:	◯Yes ◯No ◉Missing
Illicit drugs:	◯Yes ◯No ◉Missing
Family problems:	◯Yes ◯No ◉Missing
Relationship problems:	◯Yes ◯No ◉Missing
Grief:	◯Yes ◯No ◉Missing
Stress:	◯Yes ◯No ◉Missing
Identified Problems:	
To complete an Outcome Tool (K10), click the following	Outcome Tool (K10)
Outcome Tool (K10) score:	
**Skin**	

Skin:	Any skin problems	◯Yes ◯No ◉Missing
	Lesion to check	◯Yes ◯No ◉Missing
	Rash unspecified	◯Yes ◯No ◉Missing
	Scabies	◯Yes ◯No ◉Missing
	Pus	◯Yes ◯No ◉Missing

	Fungal	Yes No Missing
	Eczema/Allergies	Yes No Missing
	Skin cancer	Yes No Missing
	Psoriasis	Yes No Missing
	Other	Yes No Missing
Identified Problems:		

WOMEN'S HEALTH

Urinary Problems:	Yes No Missing	
Identified Problems:		
Pap smear due:	Yes No Missing	Given<br>Declined
Last done:		
Mammogram due:	Yes No Missing	
Last done:		
Contraception required:	Yes No Missing	
	Condoms OCP Mini Pill Depo Implanon Mirena Other IUD Other Missing	
Comment:		

**Sexual Health Check**	
Any problems?	Yes No Missing
Symptoms or concerns?	
Chlamydia and Gonorrhea screening advised:	Yes No Missing
Hepatitis B, HIV, Syphilis screening advised:	Yes No Missing
Have you had any unsafe sex in the last year?	Yes No Missing
**Hepatitis C Risks**	
Exposure to Hepatitis C	Yes No Missing

Tattoos	◯ Yes ◯ No ◉ Missing	
IVDU	◯ Yes ◯ No ◉ Missing	
Incarceration history	◯ Yes ◯ No ◉ Missing	
Investigation and Advice undertaken		
**Client's Overall Health Status**	◯ Very Good<br>◯ Good<br>◯ Fair<br>◯ Poor<br>◯ Very Poor<br>◉ Missing	
Current health problems:		
Current medications (include OTC medication and medication from other doctors)		
**Community and Family**		
Family Medical History		
Family Issues:		
Do you care for someone?	◯ Yes ◯ No ◉ Missing	◯ Adult ◯ Child ◉ Missing
Number of children:		
Ages of children:		
Are you cared for by someone else?	◯ Yes ◯ No ◉ Missing	
**Environmental and Living Conditions**		
Identified problems:	Homelessness	◯ Yes ◯ No ◉ Missing
	Overcrowding	◯ Yes ◯ No ◉ Missing
	Conflict at home	◯ Yes ◯ No ◉ Missing
	Other	◯ Yes ◯ No ◉ Missing
	Please specify:	
Employment Status:	Employed full-time	◯ Yes ◯ No ◉ Missing
	Employed part-time	◯ Yes ◯ No ◉ Missing
	Voluntary work	◯ Yes ◯ No ◉ Missing
	Unemployed	◯ Yes ◯ No ◉ Missing
	Study full-time	◯ Yes ◯ No ◉ Missing

	Study part-time	◯ Yes ◯ No ◉ Missing
	Carer	◯ Yes ◯ No ◉ Missing
	Home duties	◯ Yes ◯ No ◉ Missing
	Disability pension	◯ Yes ◯ No ◉ Missing
	Other pension	◯ Yes ◯ No ◉ Missing
	Casual	◯ Yes ◯ No ◉ Missing
	Contract Work	◯ Yes ◯ No ◉ Missing

Medical History/Examination by GP

New diagnosis from this health check:	◯ Yes ◯ No ◉ Missing
If Yes, please specify:	

Accidents and Injuries

Have you had any accidents (including motor vehicle) that have required hospital or GP review since last health check?	◯ Yes ◯ No ◉ Missing
If Yes, please specify:	

Medication Review

Medication Review:	◯ Yes ◯ No ◉ Missing
Medication Compliance:	◯ Takes most doses<br>◯ Takes some doses<br>◯ Does not take meds<br>◉ Missing
Identified Problems:	

Cholesterol

Cholesterol / Trig:	/
HDL / LDL:	/
Total Cholesterol / HDL:	

Known Health Problems

	☐ Asthma	☐ Hypertension
	☐ Cerebrovascular Disease	☐ Ischemic Heart Disease
	☐ Chronic Kidney Disease	☐ Rheumatic Heart Disease
	☐ COPD	☐ Type 2 Diabetes
	☐ Dyslipidemia	

PROBLEM LIST

ACTIONS

Advice:	◯ Yes ◯ No ◉ Missing	
Comments:		
Pap Smears:	◯ Yes ◯ No ◉ Missing	
Comments:		
New Medications:	◯ Yes ◯ No ◉ Missing	
Comments:		
Immunisation:	◯ Yes ◯ No ◉ Missing	
Comments:		
Referrals:	◯ Yes ◯ No ◉ Missing	
	Referral to Optometrist?	◯ Yes ◯ No ◉ Missing
	Referral to Ophthalmologist?	◯ Yes ◯ No ◉ Missing
	Referral to physiotherapists / exercise group:	◯ Yes ◯ No ◉ Missing
	Referral to Mental Health Service:	◯ Yes ◯ No ◉ Missing
	Referral to Audiologist/Australian Hearing:	◯ Yes ◯ No ◉ Missing
	Referral to dietician:	◯ Yes ◯ No ◉ Missing
Comments:		
Other Action:	◯ Yes ◯ No ◉ Missing	
Comments:		

Sign Off

☐ **Completed?** (Select this only when Health Check is complete)

User Name ______ **Password** ______ Save

Health Check Completed by:

Signed:

Date:
