# Supplementary material for: Implementing computerised Aboriginal and Torres Strait Islander health checks in primary care for clinical care and research: a process evaluation
Source: BMC Med Inform Decis Mak. 2013 Sep 21;13:108. doi: 10.1186/1472-6947-13-108 (PMC3849740; doi:10.1186/1472-6947-13-108)
Supplement: Additional file 4 — Aboriginal and Torres Strait Islander computerised antenatal health check template. [file 1472-6947-13-108-S4.pdf]

Metro South Health Service District	UR Number	
Indigenous Antenatal Health Check	Surname	
Community Health	Other Names	
	DOB	
	Sex	Female

Queensland Government Queensland Health

An * indicates a mandatory field.

PATIENT INFORMATION

Age: *			
Phone:		Mobile:	
Child:	◯ Aboriginal ◯ Aboriginal & Torres Strait Islander ◯ Torres Strait Islander ◯ Other ◉ Missing *		
Mother:	◯ Aboriginal ◯ Aboriginal & Torres Strait Islander ◯ Torres Strait Islander ◯ Other ◉ Missing *		
Mother's Marital Status:	◯ Single ◯ Separated ◯ Married ◯ De Facto ◯ Not Stated ◉ Missing		
Consent for health assessment to be used in reasearch	◯ Yes ◯ No		
Information obtained by:			

EDUCATION LEVEL COMPLETED

Mother:	◯ Year 10 or less ◯ Year 11-12 ◯ TAFE ◯ University ◯ Not Stated ◉ Missing
Father:	◯ Year 10 or less ◯ Year 11-12 ◯ TAFE ◯ University ◯ Not Stated ◉ Missing
Comments:	
Information obtained by:	

MENSTRUAL HISTORY

L.N.M.P	Certain:	◯ Yes ◯ No ◉ Missing
	First Day of L.N.M.P	
	EDD:	
Contraception:	◯ Yes ◯ No ◉ Missing	
If Yes, please specify:		
Comments:		
Information obtained by:		

HISTORY OF PRESENT PREGNANCY

When was the first antenatal visit?			
G: P:	Date pregnancy confirmed:		K:
Presenting weight: kg	Height: cm	BMI:	Presenting BP: /
Multiple pregnancies:	◯ Yes ◯ No ◉ Missing	If yes, how many foetuses?	
Medications			

(include over the counter):	
Influenza Vaccination:	○ Yes ○ No ◉ Missing
Comments:	
Information obtained by:	

REFERRED TO

Type of Care:<br>○ Share care<br>○ Hospital<br>○ Midwife<br>○ None<br>◉ Missing	○ Indigenous Antenatal Clinic<br>○ Mainstream ANC<br>○ Ipswich<br>○ Logan<br>○ Royal Women's<br>○ Mater Mother's<br>○ Other (please state below)<br>◉ Missing<br><br>Date of first hospital visit:
Information obtained by:	

OBSTETRIC HISTORY

no	dob (dd/mm/yyyy)	gestation (k)	birth weight (g)	sex	type of delivery	complications of labour, pregnancy & puerperium	hospital	breast/bottle	baby's name	
				○ Male<br>○ Female<br>◉ Missing				○ Breast<br>○ Bottle<br>◉ Missing		Delete

Add Row

Comments:	
Information obtained by:	

HISTORY

Allergies:	
Chlamydia test:	○ Yes ○ No ◉ Missing
Gonohorrhea test:	○ Yes ○ No ◉ Missing
3rd trimester increased risk of Chlamydia:	○ Yes ○ No ◉ Missing
Pap smear:	○ Yes ○ No ◉ Missing
Due:	
Medical and Surgical history:	
Mental Health history:	
General comments:	
Information obtained by:	

ORAL HEALTH

Identified Problems:	○ Yes ○ No ◉ Missing
Dental Caries:	○ Yes ○ No ◉ Missing
Gum Disease:	○ Yes ○ No ◉ Missing

Comments:	
Information obtained by:	

FAMILY HISTORY

**Client's Family history:**	
Multiple pregnancies:	◯ Yes ◯ No ◉ Missing
Comments:	
Information obtained by:	

ACCOMMODATION

	PRESENTING	3RD TRIMESTER
Type:		
Number of people living in accommodation:		
Lives with:		
Comments:		
Information obtained by:		

FAMILY & SOCIAL CIRCUMSTANCES

	PRESENTING	3RD TRIMESTER
Sole parent	◯ Yes ◯ No ◉ Missing	◯ Yes ◯ No ◉ Missing
If < 18 years old - going to school:	◯ Yes ◯ No ◉ Missing	◯ Yes ◯ No ◉ Missing
Are you employed?	◯ Yes ◯ No ◉ Missing	◯ Yes ◯ No ◉ Missing
Partner Employed?	◯ Yes ◯ No ◉ Missing	◯ Yes ◯ No ◉ Missing
Family Support?	◯ Yes ◯ No ◉ Missing	◯ Yes ◯ No ◉ Missing
Comments:		
Information obtained by:		

VIOLENCE AT HOME AND IN THE COMMUNITY

Are you ever afraid of your partner?	◯ Yes ◯ No ◉ Missing
In the last year, has your partner hit, kicked, punched or otherwise hurt you?	◯ Yes ◯ No ◉ Missing
In the last year, has your partner put you down, humiliated you or tried to control what you can do?	◯ Yes ◯ No ◉ Missing
In the last year, has your partner threatened to hurt you?	◯ Yes ◯ No ◉ Missing
Would you like help with any of this now? (if domestic violence identified)	◯ Yes ◯ No ◉ Missing
Do you feel threatened or afraid by anyone in the community?	◯ Yes ◯ No ◉ Missing
Comments:	
Information obtained by:	

NUTRITION

Nutrition problem identified:	◯ Yes ◯ No ◉ Missing
Folic Acid Supplementation:	◯ Yes ◯ No ◉ Missing
Iodine:	◯ Yes ◯ No ◉ Missing
Iron Supplementation:	◯ Yes ◯ No ◉ Missing
Education provided:	◯ Yes ◯ No ◉ Missing
Comments:	
Information Obtained by:	

HISTORY OF CAFFEINE

Caffeine (coffee, tea, green tea, Red Bull, V drinks, Coke, Pepsi, iced coffee):	◯ Yes ◯ No ◉ Missing
Drinks per day:	
Comments:	
Information obtained by:	

MATERNAL HISTORY OF TOBACCO USE

Definitions are those given by Healthy for Life and are required for data collection

Question: How often, if at all, do you now smoke cigarettes of other tobacco products?

**Category**	**Definition**	**1st Trimester < K13**	**3rd Trimester**
Daily smoker	Smokes daily	◯ Yes ◯ No ◉ Missing	◯ Yes ◯ No ◉ Missing
Weekly smoker	Smokes at least weekly but not daily	◯ Yes ◯ No ◉ Missing	◯ Yes ◯ No ◉ Missing
Irregular smoker	Smokes less than weekly	◯ Yes ◯ No ◉ Missing	◯ Yes ◯ No ◉ Missing
Ex-smoker Quit during pregnancy	Does not smoke at all now, but has smoked at least a hundred cigarettes in their lifetime and did smoke at some stage during pregnancy	◯ Yes ◯ No ◉ Missing	◯ Yes ◯ No ◉ Missing<br>If Yes, by 20 weeks<br>◯ Yes ◯ No ◉ Missing
Ex-smoker Quit before pregnancy	Does not smoke at all now, but has smoked at least a hundred cigarettes in their lifetime and did not smoke at all during pregnancy	◯ Yes ◯ No ◉ Missing	◯ Yes ◯ No ◉ Missing
Non-smoker	Does not smoke now and has smoked fewer than 100 cigarettes in their lifetime	◯ Yes ◯ No ◉ Missing	◯ Yes ◯ No ◉ Missing
Comments:			
Information obtained by:			

PATERNAL HISTORY OF TOBACCO USE

**Smoking**	
Current Smoker?	◯ Yes ◯ No ◉ Missing
Comments:	
Information obtained by:	

MATERNAL HISTORY OF ALCOHOL USE

Definitions are those given by Healthy for Life and are required for data collection

Question: At any time during your pregnancy have you consumed alcohol? If Yes: How frequently?

**Category**	**Definition**	**1st Trimester < K13**	**3rd Trimester**
High 1	Over a week has more than 7 standard drinks	◯ Yes ◯ No ◉ Missing	◯ Yes ◯ No ◉ Missing

High 2	On any day more than 2 standard drinks	◯Yes ◯No ◉Missing	◯Yes ◯No ◉Missing
Low	Over a week has less than 7 standard drinks AND on any one day no more than 2 standard drinks (spread over at least 2 hours)	◯Yes ◯No ◉Missing	◯Yes ◯No ◉Missing
No Alcohol	A person who does not drink at all during pregnancy	◯Yes ◯No ◉Missing	◯Yes ◯No ◉Missing
Comments:			
Information obtained by:			

PATERNAL HISTORY OF ALCOHOL USE

**Alcohol**	
Alcohol 0-4 standard drinks per day	◯Yes ◯No ◉Missing
Alcohol > 4 standard drinks per day	◯Yes ◯No ◉Missing
Alcohol free days (at least 2) per week	◯Yes ◯No ◉Missing
Binge drinking	◯Yes ◯No ◉Missing
Comments:	
Information obtained by:	

MATERNAL RECREATIONAL/PSYCHOACTIVE DRUGS

Definitions are those given by Healthy for Life and are required for data collection	
Healthy for Life have provided the illicit drugs listed below:<br>Any pharmaceutical drug (such as pain killers and tranquilisers) when used for NON-MEDICAL use	
Have you used other substances:	◯Yes ◯No ◉Missing
marijuana	◯Yes ◯No ◉Missing
barbiturates	◯Yes ◯No ◉Missing
heroin	◯Yes ◯No ◉Missing
cocaine	◯Yes ◯No ◉Missing
ecstasy	◯Yes ◯No ◉Missing
any injected drug	◯Yes ◯No ◉Missing
pain killers / analgesics	◯Yes ◯No ◉Missing
meth / amphetamines (speed)	◯Yes ◯No ◉Missing
methadone	◯Yes ◯No ◉Missing
LSD /synthetic hallucinogens	◯Yes ◯No ◉Missing
ketamine	◯Yes ◯No ◉Missing
tranquilisers/sleeping pills	◯Yes ◯No ◉Missing
steroids	◯Yes ◯No ◉Missing

other opiates (opiods)	◯ Yes ◯ No ◉ Missing
natural hallucinogens	◯ Yes ◯ No ◉ Missing
inhalants	◯ Yes ◯ No ◉ Missing

Question: How often, if at all, do you now use illicit drugs?

Category	Definition	1st Trimester < K13	3rd Trimester
Daily	Uses every day	◯ Yes ◯ No ◉ Missing	◯ Yes ◯ No ◉ Missing
Weekly	Uses at least weekly but not daily	◯ Yes ◯ No ◉ Missing	◯ Yes ◯ No ◉ Missing
Irregular	Uses less than weekly	◯ Yes ◯ No ◉ Missing	◯ Yes ◯ No ◉ Missing
Ex-user 1	Does not use at all now, but has used in the last 12 months and did use at some stage during pregnancy	◯ Yes ◯ No ◉ Missing	◯ Yes ◯ No ◉ Missing
Ex-user 2	Does not use at all now, and not use at any stage during pregnancy, but has used in the last 12 months	◯ Yes ◯ No ◉ Missing	◯ Yes ◯ No ◉ Missing
Non-user	Does not use now and has not used in the last 12 months	◯ Yes ◯ No ◉ Missing	◯ Yes ◯ No ◉ Missing
Comments:			
Information obtained by:			

PATERNAL RECREATIONAL/PSYCHOACTIVE DRUGS

Cannabis	◯ Yes ◯ No ◉ Missing	Amphetamines / speed	◯ Yes ◯ No ◉ Missing
Heroin	◯ Yes ◯ No ◉ Missing	Ice	◯ Yes ◯ No ◉ Missing
Methadone	◯ Yes ◯ No ◉ Missing	Chroming	◯ Yes ◯ No ◉ Missing
Cocaine	◯ Yes ◯ No ◉ Missing	Other psychoactive drugs	◯ Yes ◯ No ◉ Missing
IV injection	◯ Yes ◯ No ◉ Missing	Shared needles	◯ Yes ◯ No ◉ Missing
Prescription Drugs	◯ Yes ◯ No ◉ Missing	Non-prescription drugs	◯ Yes ◯ No ◉ Missing
Comments:			
Information obtained by:			

ULTRASOUND

Date (dd/mm/yyyy)	K	EDC (dd/mm/yyyy)	Comments	Initial	
					Delete

Add Row

URINE SCREENING

Date collected:			
Results:			
Chlamydia:	◯ Positive ◯ Negative ◉ Missing	Gonorrhoea:	◯ Positive ◯ Negative ◉ Missing
Rx (if required):	◯ Yes ◯ No ◉ Missing		
UTI Screening (MSU Result):	◯ Positive ◯ Negative ◉ Missing		
Comment:			
Information obtained by:			

LABORATORY RESULTS

K weeks	Date	Blood group	Rhesus Status	Antibody screen	Hb g/L	RPR/ TPHA	Hep B	Rubella Titre	HIV	LAB	Initials
K5-12			Positive Negative Missing	Positive Negative Missing		Positive Negative Missing	Positive Negative Missing	Positive Negative Missing	Positive Negative Missing		
K28			Positive Negative Missing	Positive Negative Missing		Positive Negative Missing	Positive Negative Missing	Positive Negative Missing	Positive Negative Missing		
K36			Positive Negative Missing	Positive Negative Missing		Positive Negative Missing	Positive Negative Missing	Positive Negative Missing	Positive Negative Missing		
Other			Positive Negative Missing	Positive Negative Missing		Positive Negative Missing	Positive Negative Missing	Positive Negative Missing	Positive Negative Missing		
Comments:											

Please initial after each result obtained

GLUCOSE TEST

Date:		Normal:	Yes No Missing
Comments:			
Please initial after completed:			

ANTENATAL EDUCATION

Topic		Date	Initials
aches and pains	Yes No Missing		
alcohol during pregnancy (incl FAS)	Yes No Missing		
breast feeding	Yes No Missing		
constipation	Yes No Missing		
diabetes	Yes No Missing		
drug use	Yes No Missing		
early discharge from hospital	Yes No Missing		
haemorrhoids	Yes No Missing		
heartburn	Yes No Missing		
itchy skin	Yes No Missing		
labour	Yes No Missing		
morning sickness	Yes No Missing		
nutrition during pregnancy	Yes No Missing		
regular antenatal checks	Yes No Missing		
sexual health	Yes No Missing		
smoking during pregnancy	Yes No Missing		
tiredness	Yes No Missing		
when to go to hospital	Yes No Missing		
Comments:			

Please initial beside each education topic

ANTENATAL VISITS DURING PREGNANCY

Date (dd/mm/yyyy)	K	BP (Systolic/Diastolic)	WT (kg)	Dysuria	WTU	FMF	FH	Oedema	Comment	Initials	
		/		◯ Yes ◯ No	Proteinuria ◯ Yes ◯ No Leucocytes ◯ Yes ◯ No			◯ Yes ◯ No			Delete

Add Row

OTHER VISITS

Date (dd/mm/yyyy)	K	Comments	Initial	
				Delete

Add Row

POSTNATAL

Intra-partum	
Labour	◯ Spontaneous<br>◯ Induced<br>◉ Missing
Type of delivery	◯ SVD<br>◯ Instrumental SVD<br>◯ Elective Caesarian<br>◯ Emergency Caesarian<br>◉ Missing
Comments:	
Information obtained by:	

BIRTH DETAILS

"

Baby Details below:			
Surname:		First(Name)	
DOB:		Place of birth:	
Baby(UR) :			
Gestational(age)	K	Weight:	g
Length:	cm	Head(Circumference)	cm
Apgar:	(1)	(5)	
Vitamin K given:	◯ Yes ◯ No ◉ Missing	Date:	
Neonatal(Screening)	◯ Yes ◯ No ◉ Missing	Date:	
Hearing Screening done:	◯ Yes ◯ No ◉ Missing	◯ Pass ◯ Fail ◉ Missing	Date:
Hepatitis B given:	◯ Yes ◯ No ◉ Missing	Date:	
Complications:			
Comments:			
**Feeding at Birth**		**Discharge Details**	
Fully breast feeding	◯ Yes ◯ No ◉ Missing	Date:	
Partially breast feeding	◯ Yes ◯ No ◉ Missing	Age:	
Formula(brand)	◯ Yes ◯ No ◉ Missing	Weight:	g
Expressed breast milk	◯ Yes ◯ No ◉ Missing		

Other	◯ Yes ◯ No ◉ Missing		
Comments:			
Information obtained by zat one:			

Add Baby

MOTHER POSTNATAL PROGRESS (FIRST VISIT POST DELIVERY)

**Discussed with patient?**		**Discussed with patient?**	
breast / nipples	◯ Yes ◯ No ◉ Missing	dysuria	◯ Yes ◯ No ◉ Missing
perineum	◯ Yes ◯ No ◉ Missing	legs	◯ Yes ◯ No ◉ Missing
abdominal wound	◯ Yes ◯ No ◉ Missing	diet	◯ Yes ◯ No ◉ Missing
lochia	◯ Yes ◯ No ◉ Missing	maternal mood	◯ Yes ◯ No ◉ Missing
fundal height (if applicable)	◯ Yes ◯ No ◉ Missing	Needs Pap Smear	◯ Yes ◯ No ◉ Missing
Contraception required?	◯ Yes ◯ No ◉ Missing		
Other	◯ Yes ◯ No ◉ Missing	Postnatal check due	◯ Yes ◯ No ◉ Missing
Comments:			
Information obtained by:			

BABY POSTNATAL PROGRESS (FIRST VISIT POST DELIVERY)

Preterm delivery:	◯ Yes ◯ No ◉ Missing
Cogenital abnormalities:	◯ Yes ◯ No ◉ Missing
infection:	◯ Yes ◯ No ◉ Missing
jaundice:	◯ Yes ◯ No ◉ Missing
Was baby admitted to Special Care Nursery / Intensive Care?	◯ Yes ◯ No ◉ Missing
other:	◯ Yes ◯ No ◉ Missing
Comments:	
Information obtained by:	

PROBLEM LIST

ACTIONS

Advice:	◯ Yes ◯ No ◉ Missing
If yes, what:	

Pap Smears:	◯ Yes ◯ No ◉ Missing
If yes, what:	
New Medications:	◯ Yes ◯ No ◉ Missing
If yes, what:	
Immunisation:	◯ Yes ◯ No ◉ Missing
If yes, what:	
Referrals:	◯ Yes ◯ No ◉ Missing
If yes, what:	
Other Action:	◯ Yes ◯ No ◉ Missing
If yes, what:	

Sign Off

☐ **Completed?** (Select this only when Health Check is complete)

User Name ______ **Password** ______ Save

Health Check Completed by:

Signed:

Date:
