# Supplementary material for: Implementing computerised Aboriginal and Torres Strait Islander health checks in primary care for clinical care and research: a process evaluation
Source: BMC Med Inform Decis Mak. 2013 Sep 21;13:108. doi: 10.1186/1472-6947-13-108 (PMC3849740; doi:10.1186/1472-6947-13-108)
Supplement: Additional file 5 — Aboriginal and Torres Strait Islander computerised diabetes health check template. [file 1472-6947-13-108-S5.pdf]

Queensland Government Queensland Health	**Metro South Health Service District**<br>**Indigenous Health Annual Diabetic Check**<br>**Community Health**	UR Number<br>Surname<br>Other Names<br>DOB<br>Sex

An * indicates a mandatory field.

Doctor's Name: *		Nurse: *	
Year of Diagnosis:		Duration of Disease: *	yrs
Date:		Registered with NDSS:	◯ Yes ◯ No ◉ Missing
Consent for health assessment to be used in research: *	◯ Yes ◯ No	NDSS Membership number if available):	
How many times have you engaged in moderate physical activity (huff and puff) in the last week (30 mins or more)?			
Comments:			
Height:	cm		
Weight:	kg		
BMI:			
**Smoking:**	Never smoked:	◯ Yes ◯ No ◉ Missing	
	Ex-smoker:	◯ Yes ◯ No ◉ Missing	
	Current Smoker:	◯ Yes ◯ No ◉ Missing	
	Wishes to quit:	◯ Yes ◯ No ◉ Missing	
	Age started:		
	Number of cigarettes / day:		
	Pack Years:		
**Alcohol**	Do you drink alcohol?	◯ Yes ◯ No ◉ Missing	
	If yes, please state if harmful or non-harmful levels	☐ > 2 std drinks/day avg; drinks 6-7 days/wk; > 14 std drinks/wk<br>☐ ≤ 2 std drinks/day, 1-2 alcohol free days/wk; <14 std drinks/wk<br>☐ > 4 std drinks on any one day<br>☑ Missing	
	Diet Control:	◯ Yes ◯ No ◉ Missing	

**Management**	Tablet:	Yes No Missing
	Insulin:	Yes No Missing
	Tablet & Insulin:	Yes No Missing
	Dietary Knowledge:	
	Diabetes Education:	
	Home Monitoring:	Yes No Missing
	Records Book:	Yes No Missing
**Blood Pressure:**	B/P LA (systolic / diastolic):	/ **Sit**<br>/ **Stand**<br>/ **Lying**
	B/P RA (systolic/diastolic):	/ **Sit**<br>/ **Stand**<br>/ **Lying**
**Foot Screen Risks:**	None:	Yes No Missing
	Foot Deformity:	Yes No Missing
	Reduce Pulses:	Yes No Missing
	Past History Foot Ulcer:	Yes No Missing
	Insensitive to Monofilament:	Yes No Missing
	Inability to Selfcare:	Yes No Missing
	Amputation:	Yes No Missing
**Waverform & ABI:**	Waveform LL:	**DP:** **PT:**
	ABI LL:	
	Waveform RL:	**DP:** **PT:**
	ABI RL:	
	Glasses Used?	Yes No Missing
	VA Right:	
	VA Left:	

**Vision:**	VA Both:	
	Retinal Photograph:	◯ Yes ◯ No ◉ Missing
	Comments:	
	Opthalmologist - last visit:	
**Pathology:**	Cholesterol / Trig:	/
	HDL / LDL:	/
	Total Cholesterol /HDL:	
	eGFR:	
	Stage of renal failure:	
	ACR	Albuminuria: mg/L
		Creatinine: mmol/L
		AC Ratio:
	HbA1C:	%
**ACOC:**	◯ Yes ◯ No ◉ Missing	
	Comments:	

**MEDICAL ASSESSMENT**			
Medication Review required?	◯ Yes ◯ No ◉ Missing		
Medication Review completed?	◯ Yes ◯ No ◉ Missing		
Aspirin:	◯ Yes ◯ No ◉ Missing		
Insulin:	◯ Yes ◯ No ◉ Missing		
BP Lowering:	◯ Yes ◯ No ◉ Missing		
Metformin:	◯ Yes ◯ No ◉ Missing		
Lipid Lowering Drug:	◯ Yes ◯ No ◉ Missing		
Angina/IHD/CHF:	◯ Yes ◯ No ◉ Missing		
TIA/CVA:	◯ Yes ◯ No ◉ Missing		

Periph Vasc Disease:	◯ Yes ◯ No ◉ Missing		

PROBLEM LIST

ACTIONS

Advice:	◯ Yes ◯ No ◉ Missing	
	If Yes, what:	
New Medications:	◯ Yes ◯ No ◉ Missing	
	If Yes, what:	
Immunisation:	◯ Yes ◯ No ◉ Missing	
	If Yes, what:	
Referrals:	◯ Yes ◯ No ◉ Missing	
	☐ Diabetes Educator ☐ Dietitian ☐ Endocrinologist ☐ Excercise Physiologist ☐ Nephrologist ☐ Opthalmologist ☐ Optometrist ☐ Podiatrist ☐ Physiotherapist ☐ Other	
	If other, please specify:	
Other Action:	◯ Yes ◯ No ◉ Missing	
	If Yes, what:	

Sign Off

☐ **Completed?** (Select this only when Health Check is complete)

User Name **Password** Save

Health Check Completed by:

Signed:

Date:
