# Supplementary material for: Implementing computerised Aboriginal and Torres Strait Islander health checks in primary care for clinical care and research: a process evaluation
Source: BMC Med Inform Decis Mak. 2013 Sep 21;13:108. doi: 10.1186/1472-6947-13-108 (PMC3849740; doi:10.1186/1472-6947-13-108)
Supplement: Additional file 6 — Aboriginal and Torres Strait Islander computerised diabetic retinopathy template. [file 1472-6947-13-108-S6.pdf]

**Metro South Health Service District**	UR Number
**Indigenous Health Diabetic Retinopathy Screening Reporting**	Surname
**Community Health**	Other Names
	DOB
	Sex

An * indicates a mandatory field.

Duration of Disease:	yrs		
HbA1C:	%		
BP:	/		
Visual Acuity:	L 6 /<br>R 6 /		
1. Adequate photograph for interpreting?	**Left Eye:**	○ Yes ○ No ◉ Missing	
	**Right Eye:**	○ Yes ○ No ◉ Missing	
2. Appearance of Fundi?		**Left Eye**	**Right Eye**
	No NDPR	☐	☐
	Mild NDPR	☐	☐
	Moderate NDPR	☐	☐
	Severe NDPR	☐	☐
	PDR	☐	☐
	Other Pathology	**Left Eye**	○ Yes ○ No ◉ Missing
		**Right Eye**	○ Yes ○ No ◉ Missing
	Diagnosis	**Left Eye**	
		**Right Eye**	
3. Presence of Diabetic Maculopathy	**Left Eye**	○ Yes ○ No ◉ Missing	
	**Right Eye**	○ Yes ○ No ◉ Missing	
4. Management Plan		**Left Eye**	**Right Eye**
	No action - review 1-2 years	☐	☐
	Routine referral to opthalmologist	☐	☐
	Urgent referral to opthalmologist	☐	☐
	Direct contact with opthalmologist	☐	☐
	Inadequate vew - repeat photography	☐	☐

Sign Off

☐ **Completed?** (Select this only when Health Check is complete)

User Name

Save

Health Check Completed by:

Signed:

Date:
