# Supplementary material for: Implementing computerised Aboriginal and Torres Strait Islander health checks in primary care for clinical care and research: a process evaluation
Source: BMC Med Inform Decis Mak. 2013 Sep 21;13:108. doi: 10.1186/1472-6947-13-108 (PMC3849740; doi:10.1186/1472-6947-13-108)
Supplement: Additional file 7 — Computerised health check user satisfaction survey. [file 1472-6947-13-108-S7.docx]

User Satisfaction Survey

Answers on a 10 point scale where 1 represented extremely dissatisfied and 10 represented extremely satisfied

How often do you think you would complete an Electronic Health Template?

How satisfied were you with the communications for the new system?

How satisfied were you with the training?

Was the training material provided easy to follow?

How satisfied were you with the IT support for the new system?

How much value have the Electronic health templates added to your work?

How easy did you find the process of entering/ changing patient information for each Template?

Please rate your overall satisfaction with the Electronic Health Templates?
